# Successful upfront combination therapy against pulmonary arterial hypertension associated with unilateral absence of the pulmonary artery: a case report

**DOI:** 10.1093/ehjcr/ytaf328

**Published:** 2025-07-10

**Authors:** Naoyuki Otani, Shoya Ono, Takushi Sugiyama, Hiroshi Harasawa, Takanori Yasu

**Affiliations:** Department of Cardiology, Dokkyo Medical University Nikko Medical Center, 145-1 Moritomo, Nikko City, Tochigi 321-1298, Japan; Department of Cardiovascular Medicine and Nephrology, Dokkyo Medical University Nikko Medical Center, 145-1 Moritomo, Nikko City, Tochigi 321-1298, Japan; Department of Cardiovascular Medicine and Nephrology, Dokkyo Medical University Nikko Medical Center, 145-1 Moritomo, Nikko City, Tochigi 321-1298, Japan; Department of Pulmonary Medicine, Dokkyo Medical University Nikko Medical Center, 145-1 Moritomo, Nikko City, Tochigi 321-1298, Japan; Department of Cardiovascular Medicine and Nephrology, Dokkyo Medical University Nikko Medical Center, 145-1 Moritomo, Nikko City, Tochigi 321-1298, Japan

**Keywords:** Pulmonary arterial hypertension, Unilateral absence of pulmonary artery, Upfront combination therapy, Case report

## Abstract

**Background:**

Unilateral absence of the pulmonary artery (UAPA), a rare congenital condition, is associated with pulmonary hypertension in 25% of cases.

**Case summary:**

A 67-year-old Japanese woman presented with UAPA and progressive severe pulmonary arterial hypertension. During hospitalization for acute coronary syndrome 8 years ago, the patient experienced apnoea. A polysomnography test confirmed obstructive sleep apnoea syndrome. Home oxygen therapy (1 L/min) and continuous night-time positive pressure breathing therapy were initiated. Dyspnoea on exertion gradually worsened 1 year ago. The estimated right ventricular systolic pressure (RVSP) on echocardiography was elevated (73 mmHg). She was urgently admitted with progressive dyspnoea (World Health Organization [WHO] class II to class IV) and marked hypoxaemia, even when receiving oxygen (3 L/min). Isolated left UAPA with severe pulmonary hypertension was diagnosed based on right heart catheterization (RHC). The patient declined continuous subcutaneous prostacyclin analogue injection. Therefore, triple therapy with macitentan (10 mg), selexipag (0.4 mg), and riociguat (3 mg) was initiated. However, RVSP remained high during the first 3 months. The selexipag dose was titrated to 3.2 mg/day over 6 months, which improved RVSP to 32 mmHg on echocardiography and the mean pulmonary artery pressure on RHC decreased to 39 mmHg. One year later, due to recurrent dyspnoea on light exertion, iloprost inhalation therapy was initiated. The patient has since progressed well, maintaining WHO class II during a 7-year follow-up period.

**Discussion:**

Prostacyclin inhalation combined with triple therapy can be an effective treatment strategy for patients with UAPA-associated pulmonary arterial hypertension.

Learning points

- Unilateral absence of the pulmonary artery (UAPA) is a rare disease, with pulmonary hypertension serving as a critical prognosis-associated factor. Although surgical treatment is used in paediatric patients, no consensus has been reached on drug therapy for pulmonary hypertension in adults, presenting a therapeutic challenge.
- Patients with severe UAPA-associated pulmonary hypertension may benefit from inhaled prostacyclin combined with triple therapy, which is the recommended strategy for drug treatment of pulmonary arterial hypertension.

## Introduction

Unilateral absence of the pulmonary artery (UAPA) is a rare congenital condition that was first identified in 1952 using angiography and cardiopulmonary testing.^1^ UAPA occurs in 1 in 200 000 adults, and pulmonary arterial hypertension (PAH) is present in a quarter of all UAPA cases. Regarding treatment, no consensus has been reached for adult patients with UAPA-associated PAH. In most patients with symptomatic PAH, drug treatment typically used for UAPA-associated PAH is effective.^2^ We present the case of a 67-year-old Japanese woman with UAPA and progressive severe PAH (World Health Organization [WHO] class IV) who responded well to upfront combination therapy comprising triple oral medication and iloprost inhalation.

## Summary figure

**Figure:**
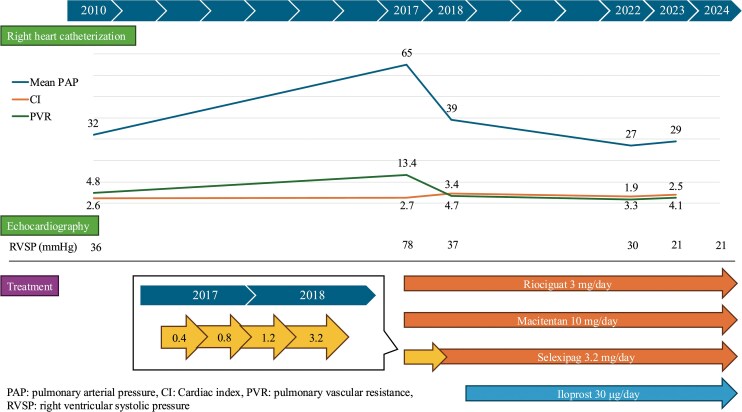


### Case presentation

**Figure 1:**
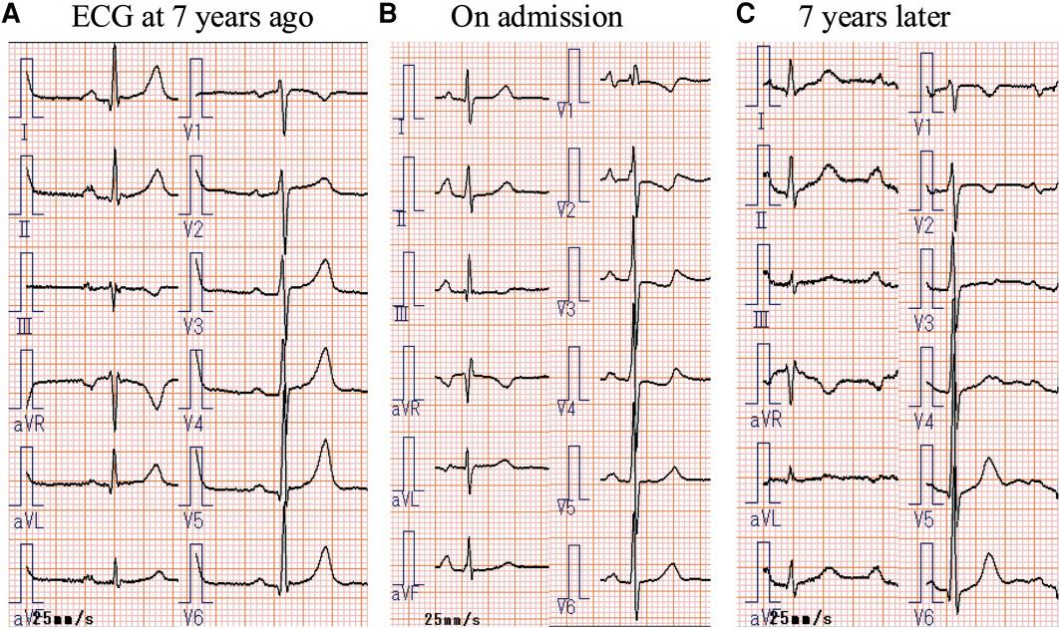
(*A*) ECG performed 7 years ago demonstrates a sinus rhythm within the normal range. (*B*) ECG obtained on admission reveals significant progression of right ventricular hypertrophy with pressure overload, characterized by the presence of an S1Q3T3 pattern, a qR pattern in lead V1, P pulmonale, and inverted T waves in leads V2−V4. (*C*) ECG manifestation of right ventricular pressure overload is remarkably resolved at 7 years after discharge by following upfront combination therapy for pulmonary arterial hypertension. ECG, electrocardiography; PAH, pulmonary arterial hypertension.

A 67-year-old Japanese woman presented with worsening dyspnoea on exertion (DOE). A left pulmonary artery defect was detected on contrast-enhanced computed tomography (CT) 17 years ago. However, the patient declined surgical treatment. She had a history of hypertension, dyslipidaemia, and diabetes mellitus and underwent percutaneous angioplasty following acute coronary syndrome 8 years ago. Transthoracic echocardiography (TTE) at that time showed an estimated right ventricular systolic pressure (RVSP) of 42 mmHg. During hospitalization, she was diagnosed with obstructive sleep apnoea syndrome, prompting home oxygen (1 L/min) and continuous night-time positive airway pressure therapy. Right heart catheterization (RHC), performed 7 years ago, showed a mean pulmonary artery pressure (PAP) of 32 mmHg. Electrocardiography (ECG) revealed sinus rhythm within normal limits (*Figure 1A*). Her condition remained stable. However, owing to a gradual worsening of DOE 1 year ago, home oxygen therapy was increased to 3 L/min. Regardless, the patient was urgently admitted with progressive dyspnoea and marked hypoxaemia (SpO₂ 87%).

**Figure 2:**
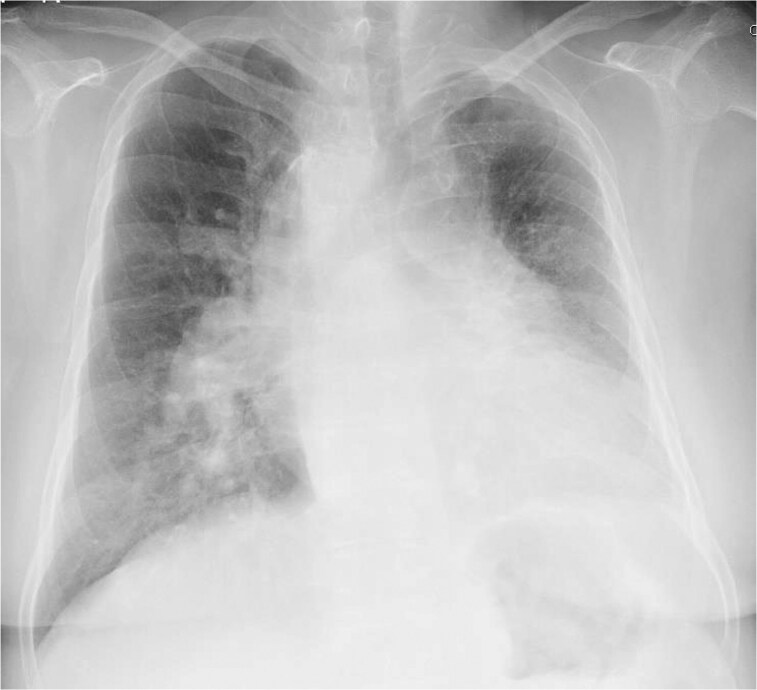
Chest radiography showing the right aortic arch and trachea deviated to the left, decreased left lung volume and right lung hyperinflation, dilated right pulmonary artery, and protrusion of the left second and fourth arches.

**Figure 3:**
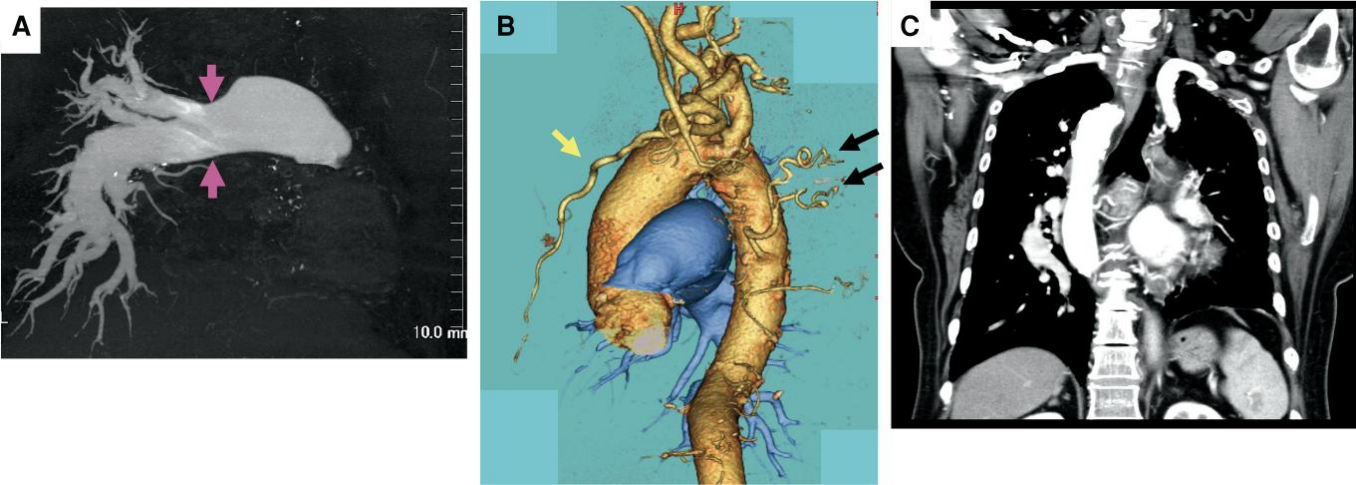
(*A*) Three-dimensional CT scan of the chest showing dilatation of the main trunk of the right pulmonary artery (arrow) and a defect from the origin of the left pulmonary artery. The right pulmonary artery is tapered peripherally but is not occluded. (*B*) The left internal thoracic artery (arrows) and left bronchial arteries (arrows) are well developed to supply blood flow to the left lung. (*C*) Coronal section of enhanced CT scan showing the right aortic arch. CT, computed tomography.

**Figure 4:**
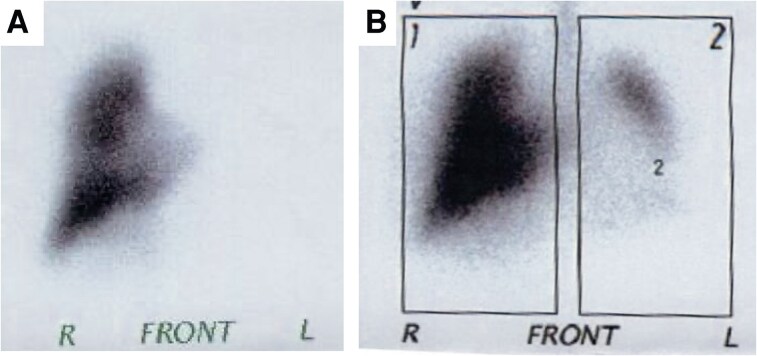
Lung scintigraphy showing no perfusion (*A*) and decreased ventilation (*B*) of the left lung.

Physical examination revealed a normal first heart sound, whereas the second heart sound had a loud pulmonic component. A grade IV/VI holosystolic murmur was audible along the left sternal border at the fourth intercostal space. She had no jugular venous distension and had bilateral pitting pedal oedema. ECG revealed sinus rhythm and features of right ventricular hypertrophy with pressure overload (*Figure 1B*). Chest radiography showed left deviation of the trachea and a dilated right pulmonary artery (*Figure 2*). Three-dimensional contrast CT revealed absence of the left and dilation of the right main pulmonary artery with distal tapering (*Figure 3A*). The left internal thoracic and bronchial arteries were well developed and supplied sufficient blood flow to the left lung (*Figure 3B*). A right arterial arch was also found, indicating congenital abnormalities in the running of blood vessels (*Figure 3C*). Lung perfusion scintigraphy revealed no perfusion and decreased ventilation in the left lung (*Figure 4*). Pulmonary function tests and high-resolution chest CT excluded chronic lung disease. Her serological workup for other associated causes of PAH was negative.

TTE revealed normal left ventricular systolic function, a flattened interventricular septum during systole, moderate tricuspid regurgitation, and a RVSP of 78 mmHg. Pulmonary angiography showed that the right pulmonary artery tapered distally but did not show reticular lesions or vascular occlusion, which are typical signs of chronic thromboembolic pulmonary hypertension. The right subclavian and brachiocephalic arteries diverged separately from the right aortic arch, and the left common carotid artery and left subclavian artery showed aortic anomalies diverging from the ascending aorta. Intracardiac and intrapulmonary shunts were excluded. RHC showed severe pulmonary hypertension with a mean PAP of 65 mmHg, pulmonary artery wedge pressure of 6 mmHg, cardiac output of 4.4 L/min, and pulmonary vascular resistance of 1075.2 dynes/s/cm^−5^ (13.4 Wood units).

Severe PAH (WHO functional class IV) and UAPA were diagnosed. The patient did not consent to continuous subcutaneous prostacyclin analogue injection. Therefore, triple combination therapy with macitentan, riociguat, and selexipag was initiated (Treatment algorithm for type 1 PAH).^3–6^ The selexipag dose was increased to 1.2 mg/day over 4 months. However, the DOE did not improve, and the estimated RVSP of 89 mmHg remained unchanged. After selexipag dose titration to 3.2 mg/day over another 2 months, DOE dramatically improved, the estimated RVSP on TTE decreased to 32 mmHg, and the mean PAP on RHC decreased to 39 mmHg. Owing to DOE recurrence 1 year later, the patient was prescribed an iloprost inhaler. Inhaled prostacyclin analogues have a relatively low incidence of systemic side effects and, compared with parenteral therapies, are easier to use.^7^ The patient responded well to treatment, with symptom improvement (WHO functional class II). She was taking macitentan 10 mg/day, riociguat 3 mg/day, selexipag 3.2 mg/day, and iloprost inhaler 30.0 µg/day 5 years later. TTE revealed a decreased estimated RVSP of 25 mmHg. RHC showed a mean PAP of 27 mmHg, pulmonary artery wedge pressure of 13 mmHg, cardiac output of 5.0 L/min, cardiac output index of 3.3 L/min/m^2^, and pulmonary vascular resistance of 155 dynes/s/cm^−5^ (1.94 Wood units), indicating marked improvement. The ECG manifestation of right ventricular pressure overload was dramatically resolved (*Figure 1C*). The patient later switched to inhaled treprostinil and, as of December 2024, lives independently at home. The patient continued the medication and had no PAH exacerbation (WHO functional class II).

## Discussion

The lack of consensus on treatments for adults with UAPA presents a significant therapeutic challenge. Surgical treatment is often successful in paediatric patients but is not favoured in adults owing to the occurrence of irreversible lung hypoplasia.^2^ No consensus exists regarding drug treatment for UAPA-associated PAH. Here, we present a case of successful treatment of severe PAH using initial triple combination therapy and inhaled iloprost.

Dyspnoea is a common symptom of PAH and occurs in >40% of affected individuals.^2^ The overall mortality rate for UAPA is ∼7%, primarily attributed to pulmonary hypertension and bleeding complications.^8^ Haemoptysis occurs due to haemorrhage into the lung parenchyma because of high arterial pressure in the bronchial arterioles and venules, which attempt to replace the absent pulmonary circulation.^9^ Haemoptysis is the presenting symptom in ∼20% of patients.^2^ However, our patient exhibited well-developed bronchial arteries without haemoptysis.

The factors that determine the presence or absence of PAH in patients with UAPA remain unclear. Elder *et al*. reported that UAPA cases exhibit an exaggerated rise in PAP during exercise.^10^ Furthermore, when part of the existing pulmonary vascular bed is temporarily removed from circulation through balloon occlusion, PAP markedly increases in patients with UAPA but not in healthy individuals.^10,11^ Although PAH can be triggered in patients with UAPA by high-altitude pulmonary oedema or pregnancy,^5^ the specific mechanism triggering PAH in this patient remains unknown.

The presence of PAH is an important prognostic factor in UAPA. A review of 108 cases in 2002 showed that 44% of patients with UAPA have PAH, which may impact their prognosis.^2^ In most patients with symptomatic PAH, conventional drug treatment yields positive outcomes.^4–6^ Vasodilator therapies, including endothelin receptor antagonists, calcium channel blockers, and intravenous prostacyclins, are typically used based on clinical presentation.^5,6^ In this case, upfront therapy with a combination of three pulmonary vasodilators for a relatively short period was effective. Moreover, introducing an iloprost inhaler significantly improved the patient’s daily activity.

In conclusion, prostacyclin inhalation combined with triple therapy represents a promising treatment strategy for patients with PAH associated with UAPA.

## Data Availability

The data underlying this article will be shared on reasonable request to the corresponding author.

## Lead author biography

Dr Naoyuki Otani is a Japanese cardiologist and clinical pharmacologist specializing in diagnostic ultrasound, heart failure, and early phase drug discovery.

## References

[1] Madoff IM, Gaensler EA, Strieder JW. Congenital absence of the right pulmonary artery; diagnosis by angiocardiography, with cardiorespiratory studies. N Engl J Med 1952;247:149–157. 14941307 10.1056/NEJM195207312470501

[2] Ten Harkel ADJ, Blom NA, Ottenkamp J. Isolated unilateral absence of a pulmonary artery: a case report and review of the literature. Chest 2002;122:1471–1477. 12377882 10.1378/chest.122.4.1471

[3] Seedat F, Kalla IS, Feldman C. Unilateral absent pulmonary artery in an adult—a diagnostic and therapeutic challenge. Respir Med Case Rep 2017;22:238–242. 28951831 10.1016/j.rmcr.2017.09.004 PMC5604951

[4] Sitbon O, Channick R, Chin KM, Frey A, Gaine S, Galiè N, et al Selexipag for the treatment of pulmonary arterial hypertension. N Engl J Med 2015;373:2522–2533. 26699168 10.1056/NEJMoa1503184

[5] Humbert M, Kovacs G, Hoeper MM, Badagliacca R, Berger RMF, Brida M, et al 2022 ESC/ERS Guidelines for the diagnosis and treatment of pulmonary hypertension. Eur Heart J 2022;43:3618–3731. 36017548 10.1093/eurheartj/ehac237

[6] Tamura Y, Kumamaru H, Satoh T, Miyata H, Ogawa A, Tanabe N, et al Effectiveness and outcome of pulmonary arterial hypertension-specific therapy in Japanese patients with pulmonary arterial hypertension. Circ J 2018;82:275–282. 10.1253/circj.CJ-17-0139 28747612

[7] Badesch DB, McLaughlin VV, Delcroix M, Vizza CD, Olschewski H, Sitbon O, et al Prostanoid therapy for pulmonary arterial hypertension. J Am Coll Cardiol 2004;43:S56–S61. 10.1016/j.jacc.2004.02.036 15194179

[8] Raja Shariff RE, Yusoff MR, Ibrahim KS, Kasim S. Delayed diagnosis of unilateral absence of pulmonary artery presenting with recurrent bronchopneumonia. CASE 2024;8:103–108. 38524981 10.1016/j.case.2023.12.004 PMC10954568

[9] Kadir IS, Thekudan J, Dheodar A, Jones MT, Carroll KB. Congenital unilateral pulmonary artery agenesis and aspergilloma. Ann Thorac Surg 2002;74:2169–2171. 12643413 10.1016/s0003-4975(02)03979-6

[10] Elder JC, Brofman BL, Kohn PM, Charms BL. Unilateral pulmonary artery absence or hypoplasia; radiographic and cardiopulmonary studies in five patients. Circulation 1958;17:557–566. 13523767 10.1161/01.cir.17.4.557

[11] Brofman BL, Charms BL, Kohn PM, Elder JC, Newman R, Rizika M. Unilateral pulmonary artery occlusion in man; control studies. J Thorac Surg 1957;34:206–227. 13449947

